# Long-Term Exposure to High Altitude Affects Conflict Control in the Conflict-Resolving Stage

**DOI:** 10.1371/journal.pone.0145246

**Published:** 2015-12-15

**Authors:** Hailin Ma, Yan Wang, Jianhui Wu, Baoxi Wang, Shichun Guo, Ping Luo, Buxin Han

**Affiliations:** 1 Key Laboratory of Mental Health, Institute of Psychology, Chinese Academy of Sciences, Beijing, China; 2 University of Chinese Academy of Sciences, Beijing, China; 3 Institute of Education and Psychology, Tibet University, Tibet, China; 4 Key Laboratory of Behavioral Science, Institute of Psychology, Chinese Academy of Sciences, Beijing, China; 5 Key Laboratory of Jiangxi Province for Psychology and Cognition Science, School of Psychology, Jiangxi Normal University, Nanchang, China; 6 Department of Psychology, Tsinghua University, Beijing, China; Liaoning Normal University, CHINA

## Abstract

The neurocognitive basis of the effect of long-term high altitude exposure on conflict control is unclear. Event related potentials (ERPs) were recorded in a flanker task to investigate the influence of high altitude on conflict control in the high-altitude group (who had lived at high altitude for three years but were born at low altitude) and the low-altitude group (living in low altitude only). Although altitude effect was not significant at the behavioral level, ERPs showed cognitive conflict modulation. The interaction between group and trial type was significant: P3 amplitude was greater in the low-altitude group than in the high-altitude group in the incongruent trial. This result suggests that long-term exposure to high altitude affects conflict control in the conflict-resolving stage, and that attentional resources are decreased to resist the conflict control in the high-altitude group.

## Introduction

In recent years, the rapid expansion of lowland Chinese population into the western part of China has led to about 6 million Han lowland immigrants living Qinghai-Tibetan Plateau permanently as of 2006 [1]. Living at such a high altitude causes a reduction of partial pressure of inspired oxygen and lower hemoglobin oxygen saturation (%SpO2), which can lead to hypoxia [2, 3], and affects cognition [4].

Long-term exposure to high altitude with hypoxia may lead to impairness on conflict control. First, conflict control function was diminished among patients suffering from hypoxia [5]. Second, previous neuroimaging studies have provided evidences of the impact of high altitude exposure on brain areas related to conflict control: the parietal lobe, prefrontal cortex (PFC), and the anterior cingulate cortex (ACC) [6–8]. Third, in the electrophysiological study, the parietal P3 component was affected by hypoxia. Specifically, the Go/NoGo and oddball tasks have shown increased P3 latency and decreased P3 amplitude among participants at high altitudes, suggesting that cognitive abilities are sensitive to high altitude [9–11]. Moreover, smaller N2 component was found in a group of people with impaired ACC functioning [12]. Therefore, conflict control may be influenced by long-term exposure to high altitude. However, the neurocognitive basis of the effect of long-term high-altitude exposure on conflict control is still unclear.

One of the most common tasks used in the literature to measure conflict is the flanker task. In this task, a target is flanked by non-target stimuli which correspond either to the same directional response as the target (congruent) or the opposite (incongruent). The flanker stimuli, which require more cognitive processing, induce conflict on incongruent trials, and incongruent flankers produce longer reaction times and reduced response accuracy than congruent trials [13].

Event-related potentials (ERPs) have high temporal resolution and can provide a window into the nature and precise temporal sequence of brain processes, making it possible to determine which stage of conflict control is affected by high altitude exposure [14, 15]. Conflict control includes different cognitive operations [16]. First, conflict detection or monitoring processing involves recognition of the conflict, evaluation of the degree of conflict, and the realization for a particular action. When the conflict is detected, resolution of conflict processing is activated [17]. ERP studies have found that in the conflict control processing N2 and P3 components are associated with conflict detection and resolution, respectively [17, 18]. The N2 peak is maximal at fronto-central electrode sites (FZ, FCZ, and CZ), and occurs in the time window between 250–350 ms post-stimulus onset [18, 19]. According to conflict monitoring theory, N2 component is related to ACC activation [20]. In healthy participants, N2 amplitude was enhanced in incongruent trials when compared to congruent trials [21, 22], which was thought to represent distinct conflict-monitoring or detection processes [17, 22, 23]. The P3 peak occurs approximately 300–500 ms after stimulus onset, and is maximal at central and parietal scalp sites (CZ and CPZ) [24, 25]. The P3 is related to the conflict resolution: larger P3 amplitude for incongruent trial is more likely to be associated with the need for a more careful evaluation of the stimulus to determine the correct response [17]. Longer P3 latency on incongruent trials is due to increased stimulus evaluation or categorization time [17, 26].

With the increase in immigrants from low altitude to high altitude, further research on the influence of high altitude in such immigrants is needed. Previous high-altitude studies explored high-altitude local residents (e.g., native Tibetans) [1, 27] and individuals with acute exposure to high altitudes [28, 29]. However, the effects of cognitive impairment on high-altitude local residents or on the acute exposure group could not generalize to the adult immigrants because genetics and other physiological adaptations of high-altitude local residents are different from low-altitude residents [1, 19]. Furthermore, physiological features (e.g., ventilatory rate, heart rate, and hematocri) of acute exposure to high altitudes were different from chronic exposure, so the influence of high altitude on cognition may have different immediate and long-term effects [30]. Therefore, studying cognitive changes in people who were born at low altitude and had lived at high altitude for a long time will advance our understanding of the effects of long-term high-altitude exposure.

In the present study, we investigated whether healthy young people who were born and raised in low altitudes, but who were then exposed to high altitude for three years, showed influence on a flanker task. The N2 components were the indicator of conflict detection, whereas the P3 was an index of conflict resolution. First, we hypothesized that there would be significant trial type effects on behavioral and ERP responses in both high and low altitude groups. Specifically, we anticipated participants would have faster and more accurate responses in congruent relative to incongruent trials, and that larger N2 amplitude and larger and later P3 component amplitude would be found in incongruent compared with congruent trials. Second, because the brain areas related to conflict control (ACC and parietal cortices) are influenced by high altitude exposure [6–8], we predicted that long-term exposure to high altitude would significantly affect conflict control processing. The effects of high altitude would be found in behavior results, with slower and less accurate responses in the high altitude group. Based on the relationship between the ACC, the parietal cortex, and the N2, P3 components [20], we expected to find smaller incongruent-N2 amplitudes and smaller and later incongruent-P3 components in the high-altitude group than the low-altitude group.

## Methods

### Participants

Forty two healthy young college students from the Han ethnic group, aged 19–24 years old, took part in this experiment. All participants signed an informed consent form before the experiment. The experiment was conducted in accordance with the Declaration of Helsinki and was approved by the Ethics Committee of the Institute of Psychology, Chinese Academy of Sciences. All participants were right-handed and had normal or corrected-to-normal vision. All participants had been born and raised in a low-altitude location (< 1,000 m). The high-altitude group consisted of twenty-one participants (9 male, 21.95 ± 1.15 years) from Tibet University. They had lived at high altitude (3,650 m) for three years, and their data was collected there. The twenty-one participants in the low-altitude group (10 male, 21.90 ± 1.67 years) had never been to a high-altitude area, and their data was collected at a low-altitude location. SpO2 was measured with a warmed hand in a resting condition using a pulse oxymeter (CMS50D, CONTEC, China).

### Procedure

**Fig 1:**
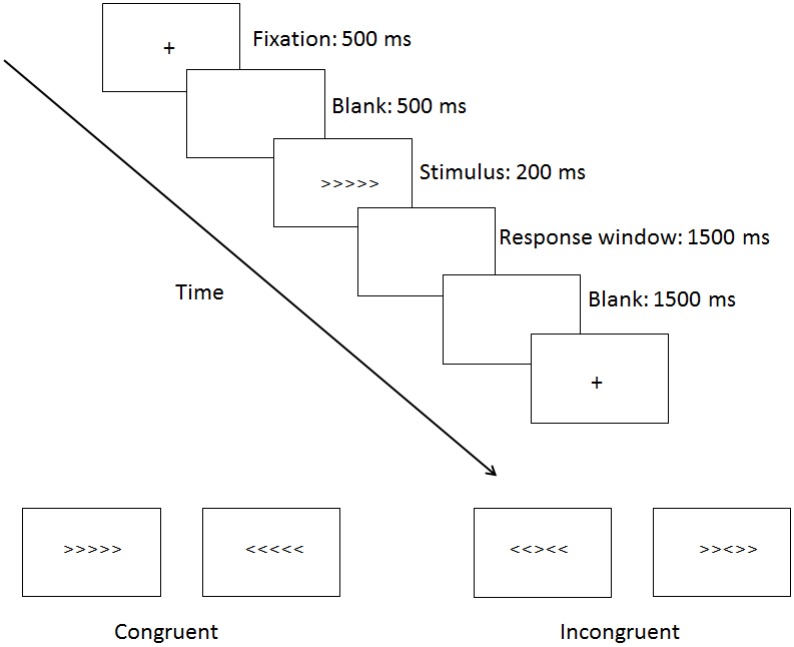
Materials and procedure. The procedure of the experimental paradigm.

In the flanker task, the following characters were used for the congruent (>>>>> or <<<<<) and incongruent (>><>> or <<><<) stimuli. The central character of the stimulus was the target, to which the participant had to make either a left or right-hand button-press, according to the direction of the arrow. A trial consisted of a fixation cross (500 ms), followed by a blank screen (500 ms), and then the stimulus (200 ms), and a 1500 ms response window. A 1500 ms blank screen followed the participant's response (or the end of the response window if no response was made), before the next trial began. There were 24 practice trials, followed by 2 experimental blocks of 80 trials, with short breaks in between blocks (Fig 1).

### ERP Recording

Electroencephalography (EEG) data were recorded from 64 scalp sites (10/20 system), using Ag/AgCl electrodes mounted in an elastic cap (Neuroscan Inc.). The physical reference electrode was approximately 2 cm posterior to CZ. Electrode impedances were kept below 5 kΩ. Vertical and horizontal electrooculogram (EOG) data were recorded from above and below the left eye and from the outer canthi of both eyes, respectively. EEG and EOG were continuously recorded at a sampling rate of 500 Hz, applying a filter bandwidth of 0.05–100Hz.

### Data Analysis

Data were analyzed with SPSS (SPSS, Inc., Chicago) for Windows. P < 0.05 was considered statistically significant. T-tests were used to analyze the SpO_2_ data with altitude group as the independent variable. Reaction times (RTs) were recorded online for all participants. Error rates and RTs for correct responses from both groups were subjected to mixed-model analysis of variance (ANOVA). The independent variable was trial type (congruent and incongruent) as within-subject factor, and altitude group (high and low) as between-subject factor.

The EEG data were re-referenced to the average of left and right mastoid (M1 and M2). Ocular artefacts were removed from the EEG signal using a regression procedure implemented with Neuroscan software [31]. The ERP data were digitally filtered with a 40 Hz low pass. Continuous EEG data was segmented into stimulus-locked (-200ms to 1000ms) ERP segments, including a 200 ms pre-stimulus baseline. Trials with various artifacts were rejected, with a criterion of ±75 μV. Waveform averages were calculated for each individual subject within each condition.

Six electrode sites (Fz, FCz, Cz, CPz, Pz, and POz) were selected for N2 and P3 data analysis. These electrodes were selected because the amplitude of both N2 and P3 was maximal by observation in these electrode sites, and previous studies have linked these regions with conflict processing [18, 32]. Mean amplitude was used for statistical analyses of the N2 and P3 components. The time windows for the N2 and P3 components were 200–350 ms and 350–650 ms, respectively. The 50 percent area latency was adopted for P3 components [33]. Mean latency was defined as the sampling point where a pre-specified fraction (50% in this case) of the total area was reached. A combination of the jack-knife method and the fractional area latency measure produced the onset latencies of the P3 wave. The amended results are reported in line with previous studies [34, 35]. Specifically, The statistical results (F-values and t-values) were corrected using the formulas: FC = F/(N-1)2, and tC = t/(N—1), where N denotes the number of observations in each condition. The specific time windows for ERP component were selected by visual inspection of ERP grand averages. Electrophysiological data were analyzed from the electrode exhibiting the largest amplitude waveform of each component of interest.

To ensure reliability of the results, the peak amplitude and peak latency were used for statistical analyses of the P3 component. The time window for P3 amplitude was 360–560ms in congruent trials and 410–610 ms for incongruent trials. We selected these specific time windows for the P3 component because P3 peak value was chose as the midpoint and the same time frame was taken for each condition.

A mixed-model ANOVA was applied to the N2 and P3 components. The ANOVA factors for the N2 and P3 components included trial type (two levels: congruent and incongruent), electrode sides (six levels: Fz, FCz, Cz, CPz, Pz, POz) as within-subject factors, and altitude group (two levels: high and low) as between-subject factor. The Greenhouse–Geisser correction was used to compensate for sphericity violations. Simple effect analyses were conducted to explore interaction effects.

Source localization of incongruent P3 amplitude values was plotted using the sLORETA [36]. The sLORETA is an efficient tool for functional mapping and source localizing; it is consistent with physiology localization. Fifty-eight electrodes (the M1, M2, two VEOG electrodes, and two HEOG electrodes were excluded from the 64 electrodes) were used for analysis. A transformation matrix was created using the electrode coordinates. The averaged waveforms in the incongruent P3 time range were converted and saved into ASCII values for both the high-altitude and low-altitude groups. sLORETA images were constructed based on the high-altitude and low-altitude group data, respectively. The version of sLORETA employed in our study was made available at http://www.unizh.ch/keyinst/NewLORETA/LORETA01.htm.

## Results

### Behavioral Results

SpO_2_was significantly higher in the low altitude group compared to the high altitude group, measuring 97.48% ± 0.93% and 90.71% ± 2.31%, respectively [*t* (1, 40) = -12.47, *p* < 0.001].

For the high-altitude and low-altitude groups, respectively, the average error rate was 1.01% ± 1.67% (mean ± S.E.) and 0.91 ± 1.12%. The average time for correct responses was 541 ± 83 ms for the high-altitude group and 527 ± 67 ms for the low-altitude group (Table 1). No significant differences were found in behavioral performance between these two groups (*p*s > 0.05). The main effect of trial type was significant for reaction time and error rate [*F* (1,40) = 328.60, *p* < 0.001; *F* (1,40) = 19.20, *p* < 0.001], with longer reaction times and more errors in incongruent trials than in congruent trials [572 ± 76 ms vs. 495 ± 72 ms; 1.79% ± 2.43% vs. 0.12% ± 0.38%].

**Table 1:** Mean reaction time (RT), errors rate and standard errors.

	RT		Errors Rate (%)	
	Congruent	Incongruent	Congruent	Incongruent
HA	504(80)	579(85)	0.18(0.46)	1.83(2.88)
LA	487(65)	567(69)	0.06(0.27)	1.75(1.96)

Mean reaction time (RT), errors rate and standard errors for high-altitude (HA) group and low-altitude (LA) group in congruent and incongruent condition.

### ERP Results

#### N2

**Fig 2:**
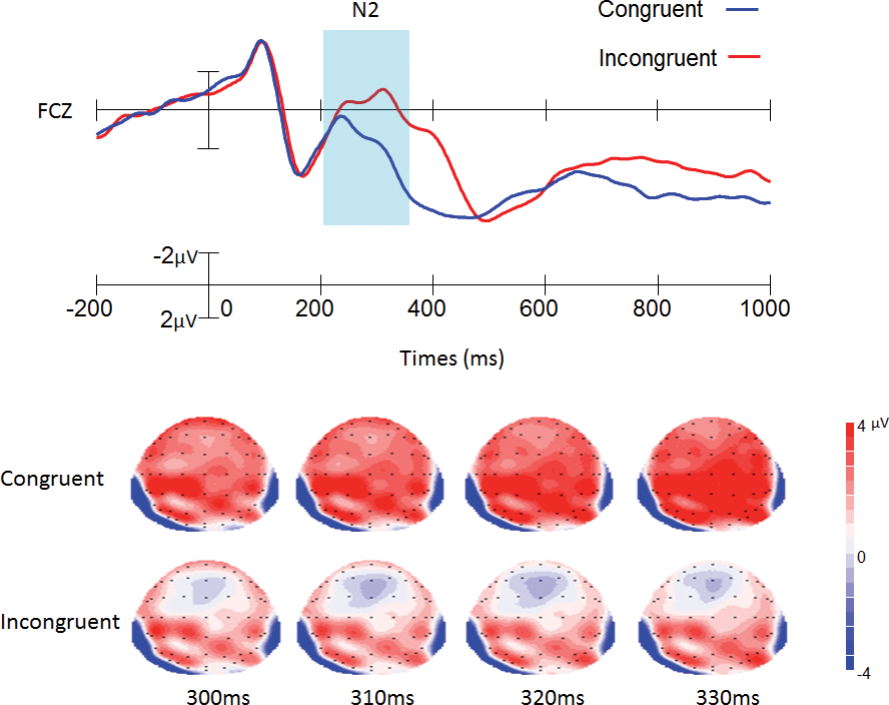
Grand average of ERP and topographical maps of N2. The grand average of ERP elicited in the congruent (blue lines) and the incongruent (red lines) conditions at Cz site. The topographical maps were generated every 10 ms from 300 ms to 330 ms. Data were averaged across 42 participants for each condition.

With regard to the amplitude of the N2 component, the main effect of trial type was significant [0.34 ± 0.55 μV vs. 1.51 ± 0.58 μV; *F* (1,40) = 19.15, *p* < 0.001], with more negative N2 amplitude in the incongruent trial than in the congruent trial for both groups (Fig 2, Table 2 and Table A in S1 File). The interaction between trial type and electrode was significant [*F* (1,36) = 4.35, *p* < 0.005], and the stimulus difference effects were maximal at the FCz site [*F* (1,40) = 26.66, *p* < 0.001].

**Table 2:** Mean amplitudes of N2 component.

	Congruent		Incongruent	
	HA	LA	HA	LA
Fz	1.12(0.85)	1.85(0.77)	-0.53(0.70)	0.58(0.76)
FCz	0.77(0.82)	2.30(0.83)	-1.05(0.75)	0.61(0.75)
Cz	0.85(0.75)	2.78(0.80)	-0.68(0.68)	1.37(0.69)
CPz	1.18(0.77)	2.92(0.89)	0.11(0.68)	1.95(0.80)
Pz	0.77(0.76)	2.07(1.03)	-0.04(0.78)	1.27(0.97)
POz	-0.24(0.68)	1.78(0.80)	-0.81(0.77)	1.32(0.83)

N2 mean amplitudes in μV (SE) during the congruent and incongruent conditions, separated between low-altitude (LA) and high-altitude (HA) group, at six midline electrodes (Fz, FCz, Cz, CPz, Pz, POz).

#### P3

**Fig 3:**
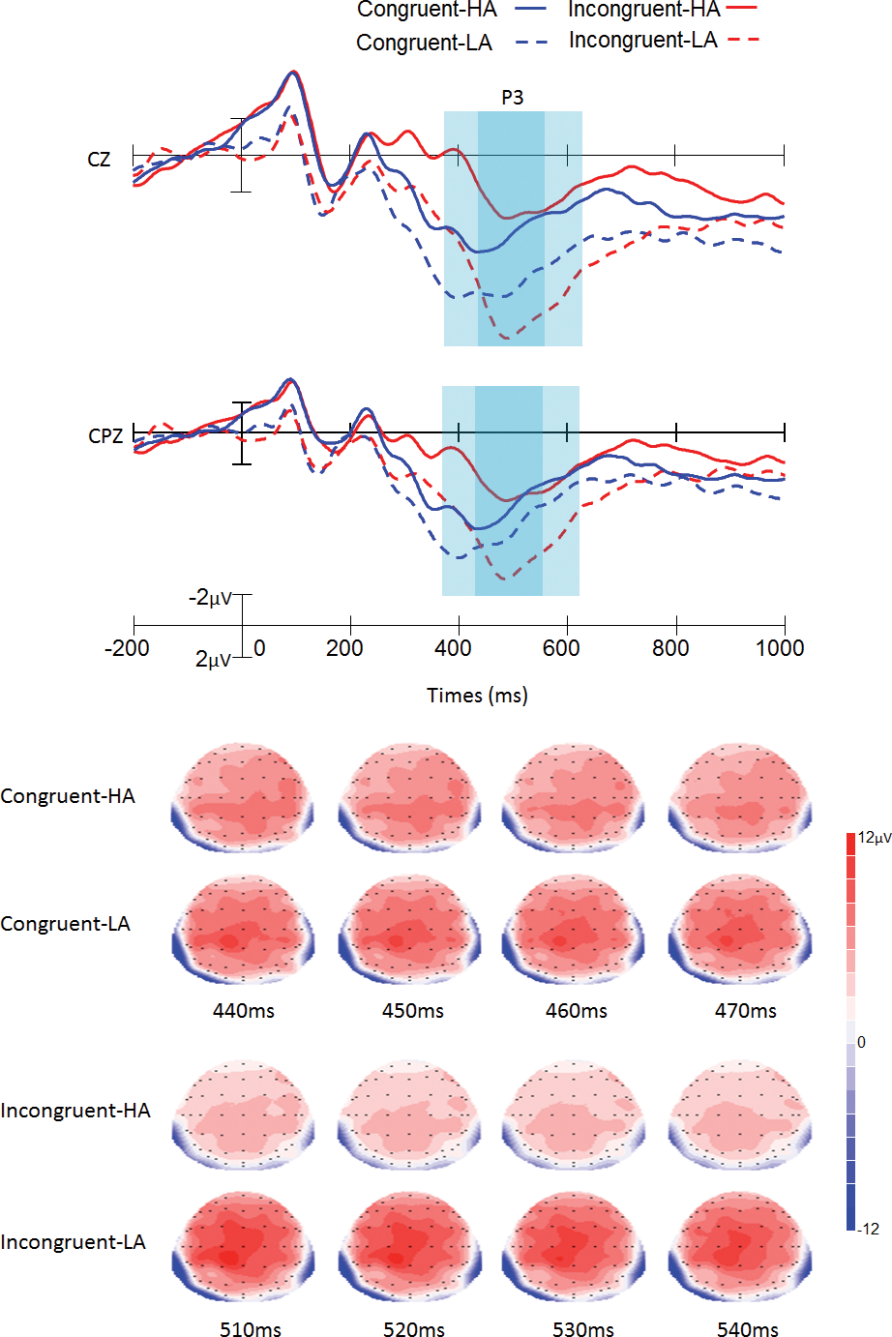
Grand average of ERP and topographical maps of P3. The grand average of ERP elicited in the low-altitude group (LA, dotted lines) and the high-altitude group (HA, solid lines) at the Cz and CPz in the congruent (blue lines) and incongruent (red lines) conditions. The topographical maps were generated every 10 ms from 440 ms to 470 ms in congruent condition, and 510 ms to 540ms in incongruent condition. Data were averaged across 21 participants for each group.

The peak amplitude and peak latency were used for statistical analyses of the P3 component. With regard to the amplitude of the P3 component, the main effect of group was significant, with smaller P3 amplitude for the high-altitude group than for the low-altitude group [2.75 ± 0.87 μV vs. 5.42 ± 0.97 μV; *F* (1,40) = 5.53, *p* < 0.05]. The interaction between trial type and group was significant [*F* (1,40) = 10.42, *p* < 0.005], with smaller P3 amplitude in the high-altitude group than in the low-altitude group under the incongruent trial [1.99 ± 0.77 μV vs. 5.76 ± 0.92 μV; *F* (1,40) = 9.87, *p* < 0.005] (Table 3, Fig 3 and Table B in S1 File). No other main effects or interactions were significant for P3 amplitude. With regard to the P3 latency, the main effect of trial type was significant, with longer P3 latency for incongruent than for congruent stimuli [507.42 ± 3.69 ms vs. 461.91 ± 3.62 ms; *F* (1,40) = 214.56, *p* < 0.001] (Table 3, Fig 3 and Table C in S1 File). No other main effects or interactions were significant.

**Table 3:** The peak amplitude and peak latency of the P3 component.

	Amplitudes				Latencies			
	Congruent		Incongruent		Congruent		Incongruent	
	HA	LA	HA	LA	HA	LA	HA	LA
Fz	2.96(0.95)	4.14(0.83)	1.19(0.88)	4.86(0.87)	455.52(3.07)	469.62(4.20)	504.10(3.65)	509.81(3.48)
FCz	3.85(0.98)	6.15(1.01)	1.87(0.90)	6.88(1.09)	458.95(3.30)	471.05(4.04)	505.62(3.77)	508.95(3.56)
Cz	4.30(0.98)	7.00(1.02)	2.57(0.92)	7.94(1.14)	462.19(3.67)	468.76(4.15)	505.33(3.74)	508.00(3.45)
CPz	4.90(0.91)	6.61(0.93)	3.42(0.89)	7.48(1.04)	456.48(3.15)	465.24(4.05)	504.57(3.72)	509.14(3.83)
Pz	3.83(0.84)	4.63(0.94)	2.49(0.84)	5.12(1.02)	454.29(2.94)	464.19(3.96)	506.57(3.83)	508.57(3.82)
POz	1.22(0.70)	1.91(0.80)	0.38(0.64)	2.30(0.89)	454.86(3.01)	461.81(3.86)	507.71(3.62)	510.67(3.81)

P3 peak amplitudes in μV (SE) and peak latencies in ms (SE) during the congruent and incongruent conditions, separated by low-altitude (LA) and high-altitude (HA) group, at six midline electrodes (Fz, FCz, Cz, CPz, Pz, POz).

The mean amplitude and the 50 percent area latency were used for the P3 component. With regard to the amplitude of the P3 component, the main effect of group was significant, with smaller P3 amplitude for the high-altitude group than for the low-altitude group [2.18 ± 0.84 μV vs. 4.70 ± 0.89 μV; *F* (1,40) = 5.75, *p* < 0.05]. The interaction between trial type and group was significant [*F* (1,40) = 6.67, *p* < 0.05], with smaller P3 amplitude in the high-altitude group than in the low-altitude group under the incongruent trial [1.44 ± 0.79 μV vs. 4.91 ± 0.96 μV; *F* (1,40) = 9.42, *p* < 0.005] (Table 4, Fig 3 and Table D in S1 File). No other main effects or interactions were significant for P3 amplitude. With regard to the P3 latency, the main effect of trial type was significant, with longer P3 latency for incongruent than for congruent stimuli [504.19 ±0.21 ms vs. 460.58 ±0.16 ms; *F* (1,40) = 110402.28, *p* < 0.001] (Table 4, Fig 3 and Table E in S1 File). No other main effects or interactions were significant.

**Table 4:** The mean amplitude and the 50 percent area latency of the P3 component.

	Amplitudes				Latencies			
	Congruent		Incongruent		Congruent		Incongruent	
	HA	LA	HA	LA	HA	LA	HA	LA
Fz	2.38(0.95)	3.90(0.73)	0.62(0.85)	3.98(0.81)	473.14(0.45)	472.57(0.34)	518.95(0.38)	519.24(0.29)
FCz	3.46(0.96)	5.77(0.89)	1.24(0.85)	5.78(1.00)	485.24(0.29)	484.86(0.30)	522.19(0.34)	522.38(0.33)
Cz	3.82(0.98)	6.39(0.89)	1.90(0.86)	6.83(1.06)	480.38(0.26)	480.19(0.19)	516.29(0.29)	516.48(0.24)
CPz	4.18(0.89)	5.71(0.80)	2.75(0.82)	6.50(1.01)	465.71(0.25)	465.71(0.25)	504.38(0.30)	504.76(0.26)
Pz	3.08(0.83)	3.77(0.85)	1.97(0.77)	4.37(1.01)	448.48(0.36)	448.48(0.27)	494.29(0.37)	494.76(0.29)
POz	0.67(0.68)	1.37(0.74)	0.15(0.59)	1.99(0.87)	410.95(0.30)	411.24(0.22)	468.19(0.66)	468.38(0.33)

P3 mean amplitudes in μV (SE) and 50 percent area latencies in ms (SE) during the congruent and incongruent conditions, separated by low-altitude (LA) and high-altitude (HA) group, at six midline electrodes (Fz, FCz, Cz, CPz, Pz, POz).

### Source Localization

**Fig 4:**
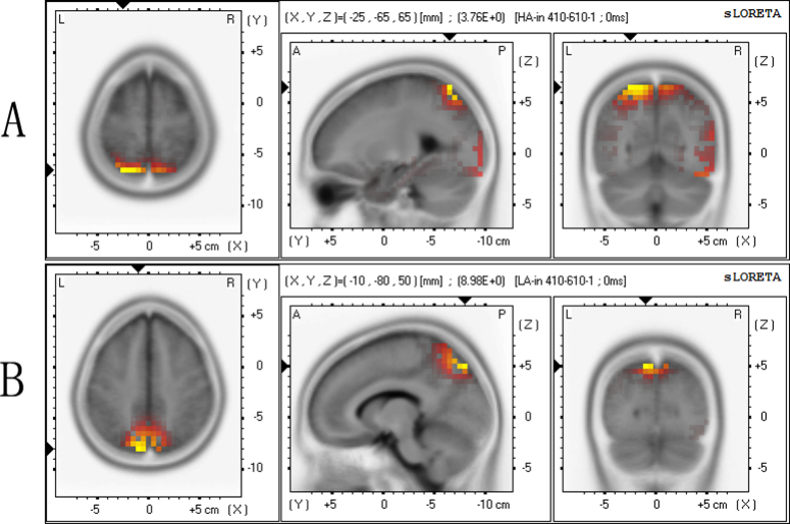
Source localization. The source localization of the surface incongruent P3 amplitude sLORETA images showing the standardized current density maxima for the high-altitude group (A) and low-altitude group (B), as seen from the horizontal, sagittal, and coronal sections. Talairach coordinates (X, Y, Z) are indicated, the activity is colour-coded. Yellow colour indicates local maxima of the incongruent P3 component is in the parietal lobe (Brodmann area 7) in the high- and low-altitude groups.

The sLORETA showed the strongest activation in the parietal lobe (Brodmann area 7) in both groups. Fig 4 shows the mean activation levels of the incongruent P3 components in the brain regions of the two groups as plotted by standardized low resolution electromagnetic tomography analysis (sLORETA). The talairach coordinates were value = 3.76E+0, (X = -25, Y = -65, Z = 65) for the high-altitude group, and value = 8.98E+0, (X = -10, Y = -80, Z = 50) for the low-altitude group.

## Discussion

Our study investigated the cognitive impact of long-term exposure to high altitude on healthy young people during a flanker task. The results showed that a conflict control effect was successfully elicited, as reflected by behavior and ERP results, with longer reaction time, higher error rates, larger N2 amplitude, and longer P3 latency in the incongruent trials than in the congruent trials. Our test focused on the neural mechanisms responsible for conflict control after long-term exposure to high altitude. SpO_2_ decreased in the high altitude group, which suggests that the subjects were suffering from hypoxia. The interaction between trial type and group was significant on P3 amplitude, with smaller P3 amplitude in the high altitude group than in the low altitude group under incongruent trials.

The main effect of trial type was significant in behavioral and ERP results. For the behavioral result, consistent with previous studies [13, 19], longer reaction times and higher error rates were found in incongruent trials compared to congruent trials, which suggested that conflict control had occurred. In line with previous ERP studies, the two typical conflict-related ERP components were detected in the present study [17, 22, 23]. The N2 amplitude was higher in the incongruent trials than in the congruent trials, reflecting the process of conflict detection [17, 22]. In addition, the maximal N2 amplitude was at FCz, and the N2 amplitude at the scalp distribution was consistent with previous studies that employed the flanker task [17, 19], suggesting that N2 may be located in the ACC and reflect conflict detection [20]. Moreover, longer P3 latency was found in the incongruent trials than in the congruent trials. According to previous studies, longer P3 latency in incongruent trials is due to increased stimulus evaluation or categorization time to determine the correct response and occurrs within the parietal cortex [17, 37]. Same as in previous research, our results show this process is delayed approximately 40 ms in incongruent compared with congruent trials [17].

The major finding of our study was that the smaller P3 amplitude was found in the high-altitude group under incongruent trials, which suggested that the conflict-resolving stage of conflict processing was significantly affected by high altitude, and there may be a decrease in control on the conflict resolution in the high-altitude group [38]. According to a previous study, in the conflict resolution stage, greater P3 amplitude is likely to be associated with more careful evaluation of the stimulus [17]. The decreased P3 amplitude in the high-altitude group may suggest that the high-altitude group could not evaluate the stimulus as well as the low-altitude group. Moreover, the flanker P3 was related to the attentional resources needed for improved cognitive control, with lower P3 amplitude seen in the less attentional resources condition [39, 40]. The same task demanded more cognitive resources from the high-altitude group than from the low-altitude group to resolve the conflict. The high cognitive demand in the high-altitude group limited the attentional resources necessary to resist the conflict control [25] and as a consequence, lower P3 amplitude was found in the high-altitude group [41]. The decreased P3 amplitude in the high-altitude group was also found in our previous study, suggest that long-term high-altitude exposure leads to diminished availability of attentional resources [42]. Using sLORETA, we localized the incongruent P3 component in the parietal cortex (Fig 4) [36]. These scalp maps further highlight the scalp distribution of the P3 amplitude modulation. This distribution is compatible with conflict resolution sources, and the parietal cortex is influenced by chronic hypoxia [6, 17, 24, 25], which may explain why lower P3 amplitude was found in the high-altitude group than in the low-altitude group under incongruent trials. However, inconsistent with our hypothesis, compared with the low altitude group, smaller incongruent-N2 amplitude was not found in high-altitude group which may suggest that the conflict detection or motoring processing in the flanker task was not affected by long term high altitude exposure.

Significant group differences were not found in the behavior results. The disappearance of the effects on behavior after prolonged exposure to high altitude may result from adaptation supported by a compensatory mechanism, which was also found in our previous study [42]. To finish the same task, the attentional resources in the high-altitude group were partly deprived to compensate the cognitive deficiency, which was reflected by lower P3 amplitude [25]. Moreover, the compensatory mechanisms lead to no group difference on P3 latency, which had strong association with the behavioral result. In our previous study, the group difference was more remarkable in the more difficult condition [42], so the group difference may become notable if the task was more difficult.

The limitations of our study are as follow: First, consequences of living at a high altitude may be influenced not only by hypoxia, but also by many other factors (e.g., differences in climate or culture). Although the participants in our study had lived in high altitude for three years and had acclimated to the environment, results should still be interpreted cautiously. Second, the task we used here was not difficult enough to clearly show differences between the two groups on behavioral results. Further research should increase the degree of task difficulty.

In conclusion, our study demonstrates that chronic exposure to high altitude alters conflict control processing at the conflict-resolving stage, as evidenced by the incongruent-lower P3 amplitude in the high altitude group. The attentional resources available to process stimuli in the function of conflict control are decreased in the high altitude group.

## Supporting Information

S1 File The ERP data of N2 and P3 components. The N2 mean amplitude during the congruent and incongruent conditions, separated by low-altitude and high-altitude group, at six midline electrodes (Fz, FCz, Cz, CPz, Pz, POz) (Table A). The P3 peak amplitude (Table B), peak latency (Table C), mean amplitude (Table D) and the 50 percent area latency (Table E) during the congruent and incongruent conditions, separated by low-altitude and high-altitude group, at six midline electrodes (Fz, FCz, Cz, CPz, Pz, POz).
(XLSX)
